# Unintended Creation or Insertion of Antisense Promoter Motifs During Codon Optimization: A Cyber-Biosecurity Risk

**DOI:** 10.3390/microorganisms14030638

**Published:** 2026-03-12

**Authors:** Elad Carmi, Roni Glikman, Yuval Dorfan

**Affiliations:** Faculty of Electrical Engineering, H.I.T.—Holon Institute of Technology, Holon 5810201, Israel

**Keywords:** codon optimization, antisense promoter, cyber-biosecurity, motif insertion, synthetic biology, DNA design, *E. coli*, translation efficiency, codon bias

## Abstract

Codon optimization is a cornerstone technique in synthetic biology and biotechnological production, aimed at enhancing heterologous protein expression through synonymous codon substitutions. While optimization traditionally focuses on forward-strand translation efficiency, its impact on the complementary DNA strand is not always carefully examined. In this study, we investigate whether codon optimization inadvertently introduces antisense motifs, specifically bacterial antisense promoter (e.g., “TATAAT”), and whether such motifs can be silently inserted into coding sequences on purpose without altering protein output. We developed a computational pipeline that (i) scans optimized sequences for antisense motifs. These could be either natural or synthetic unintended motifs; (ii) implements a silent insertion algorithm that preserves amino acid sequence; and (iii) evaluates insertion feasibility across a large genomic dataset. These components can also lead to useful scanning of synthetic sequences, before they are synthesized or ordered. It has the potential to save a great deal of time and money that might be spent in wet labs that are using the wrong sequences. Their experiments often fail due to predictable reasons, while these failures can be avoided using the software (SW) we developed, which is published here as an open source for academic and industrial usage. In a dataset of 484,741 protein-coding sequences, only 4.8% naturally contained the motif, yet 77.28% of motif-free sequences permitted silent insertions. We extend these findings with codon bias analysis, derive analytical bounds for insertion complexity, and propose computational defense strategies. These results uncover a novel cyber-biosecurity vulnerability in DNA design pipelines, emphasizing the need for bi-directional screening in codon optimization tools.

## 1. Introduction

DNA synthesis has changed rapidly in the past few decades [1], opening new opportunities like synthetic biology [2], alongside new challenges. The field of molecular biology has seen significant progress in optimizing genetic sequences [3]. Codon optimization exploits degeneracy in the genetic code to tailor DNA sequences for improved expression in heterologous hosts [4,5,6]. The method adjusts synonymous codon frequencies to match host codon usage bias, thereby increasing protein yield and translational efficiency [7,8,9,10]. Such approaches underpin major applications in gene therapy, vaccine development, and industrial biotechnology, including mRNA vaccines and recombinant protein production systems.

Historically, codon optimization algorithms prioritize metrics such as the Codon Adaptation Index (CAI) [11], GC content, and the minimization of unfavorable mRNA secondary structure [12]. Advances include deep learning models that capture sequential codon context for refined optimization [13,14,15]. Despite these improvements, most tools remain insensitive to potential functional motifs introduced on the complementary strand, particularly those that could act as antisense promoters. We focus on *E. coli* as a case study, since it is a well-known model organism [16,17], but the same SW tools and conclusions apply to all other organisms.

Promoter motifs such as the bacterial “−10” element “TATAAT” are critical regulatory elements for transcription initiation [18]. When observed in reverse complement (“ATTATA”) [19], they may function as unintended antisense promoters. Although motif engineering has been considered in some codon design contexts, the explicit risk of inadvertently creating antisense promoters and other motifs has not yet been systematically characterized. Moreover, many free and commercialized codon optimization tools are widely used as things stand, although they do not always scan for many relevant motifs. These tools’ typical users, average biologists, are often naïve and tend to trust the output sequence blindly.

The convergence of synthetic biology and cybersecurity has given rise to a new interdisciplinary domain: cyber-biosecurity [3,20,21,22,23]. Within this space, codon optimization emerges not only as a tool for improving protein expression but also as a potential vector for unintended regulatory behavior and malicious exploitation. As the capacity to synthesize and edit DNA becomes more accessible and codon optimization is increasingly performed using automated tools, the potential for errors or security loopholes grows. The rise in cyber-biosecurity highlights threats at the intersection of information security and biology. Synthetic DNA can encode harmful elements—not only biological agents but also computational payloads, like those used for SW storage [24]. While most research focuses on SW and hardware vulnerabilities, the prospect of intentional manipulation of biological regulatory elements via sequence design remains understudied. Another important factor is the flourishing of Artificial Intelligence (AI) [9]. The usage of machine learning to detect anomalies in synthetic DNA can be very useful. By training classifiers on the statistical properties of natural versus synthetic sequences, even subtle deviations introduced during optimization could be detected computationally. A key contribution of that work is the demonstration that synthetic DNA could be engineered to exploit vulnerabilities in bioinformatics SW, including buffer overflows triggered during sequence parsing. Their proof-of-concept attack transformed a DNA sequence into a vehicle for remote code execution, blurring the line between biological and computational domains. From a methodological standpoint, their work reinforces the necessity of anomaly detection algorithms in bioinformatics pipelines.

At a broader conceptual level, Elgabry et al. [3] framed synthetic biology as a potential enabler of novel forms of cybercrime. Their review presented a criminological perspective on technologies such as gene editing, bio-malware, and antisense-driven payloads, outlining scenarios where biological material could be used to circumvent security protocols or mislead forensic analysis. They emphasized the importance of developing preemptive security measures grounded in both biology and criminology. Beyond identifying threats, some researchers have explored how biological principles can inform computational defense mechanisms [25]. They proposed a bio-inspired framework for intrusion detection in network security, in which digital data was encoded as amino acid sequences and analyzed using models adapted from molecular biology. Their approach leveraged the inherent pattern recognition capabilities of bioinformatics tools to improve detection accuracy in cybersecurity contexts. Although not directly related to DNA manipulation, this methodological crossover is significant. It suggests that tools developed for motif detection and codon analysis in synthetic biology can be repurposed or adapted to other domains, and vice versa.

**Figure 1:**
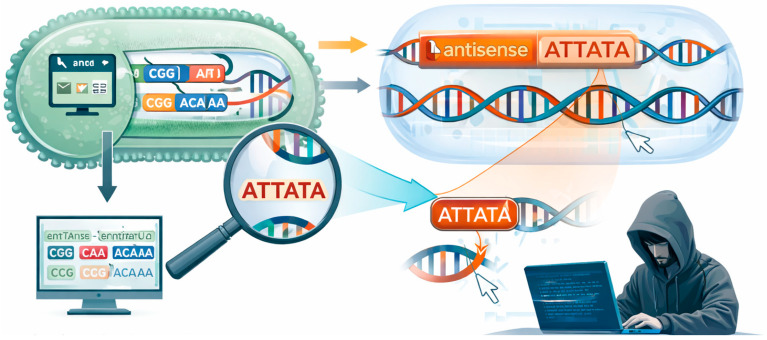
Web-based codon optimization SW can suffer from either unintended or silent insertion of harmful motifs, like antisense promoters. The SW naïve user (or even a more sophisticated one) might not identify problematic DNA sequences.

In this work, we address two questions: (i) Does codon optimization inadvertently generate antisense promoter motifs? (ii) Can such motifs be deliberately inserted into coding sequences without altering the encoded protein? In Figure 1, we present a computational framework that examines both the biological and security implications of codon redesign, supported by quantitative data and analytical modeling.

We present various simulations performed using codon optimization tools available online, both open-source and commercial. In addition, we tested and calculated the probability of various motifs in DNA sequences. Our delivery is an application that contains several testing options for customers, including comparing sequences between different optimization tools, searching for problematic motifs, and performing attempts to insert promoter motifs into the complementary strand without changing the original protein sequence.

## 2. Materials and Methods

### 2.1. The Promoter “−10” Motif: An Example Used for Our Proof of Concept (POC)

Promoters play a critical regulatory role in gene expression by controlling when, where, and to what extent a gene is transcribed. Promoters typically contain specific motifs [17] recognized by transcriptional machinery. In bacteria, a promoter consists of:

1. The −10 Region: A sequence positioned ~10 nucleotides upstream, which is crucial for DNA unwinding and the formation of the open transcription complex.
2. The −35 Region: A sequence located ~35 nucleotides upstream of the transcription start site, responsible for initial recognition by the RNA polymerase sigma factor.

As a case study, we focus on the “−10” motif for various reasons. Expanding the results to longer promoters and/or enhancers is straightforward.

### 2.2. Dataset Choice and Preprocessing

We compiled a dataset of 484,741 coding sequences from *E. coli* via the online database [26]. We focused on *E. coli* due to its well-characterized genome. Sequences shorter than 300 bp or containing ambiguous nucleotides were excluded. When focused on the classical “−10” motif, we looked for motif-free sequences and excluded those containing the reverse-complement motif, i.e., “ATTATA”.

### 2.3. Codon Optimization Framework: Analyzing Popular Tools’ Output Sequences

Codon optimization alters the codon usage of a gene to align with the preferred codons of the host organism [27], thereby potentially improving protein production. Different amino acids have codon usage biases, so adjusting the gene’s codons to match these biases can significantly improve expression efficiency. There are many types of optimization tools, and the algorithm by which each tool works is different. Some tools can only be used as a “black box”, meaning the customer cannot know what considerations were made in optimizing protein expression for a specific organism. These “black box” observations lead us to a limited set of conclusions, since these tools often use random seeds, meaning the optimization results for a given protein also vary with time. Time-varying seeds have only SW development reasons, but do not make sense from a synthetic biology point of view. On the other hand, there are open-source tools. Our research utilized an open-source codon optimization. We used a custom Python-based tool named Codon Optimization QA. This tool incorporates codon usage tables for the model organism of choice and optimizes sequences based on a composite score including CAI, GC content, and synonymous codon distribution. The evaluation of codon optimization tools in this study focused on two optimization tools that have been selected for evaluation:

1. Codon Transformer [15]—An open-source tool, which allows modifications to its original code if necessary.
2. Codon Optimization of Vector Builder [28]—A proprietary tool with a closed-source interface, where the user inputs the protein to be optimized and specifies the target host organism.

A toy example we ran to compare these two tools is the expression of an insulin protein whose host organism is *Homo sapiens* [29], aiming to evaluate the differences between the optimized DNA sequences for *E. coli* produced by these two tools.

### 2.4. New Quality Assurance (QA) Open-Source SW Tool for Codon Optimization

**Figure 2:**
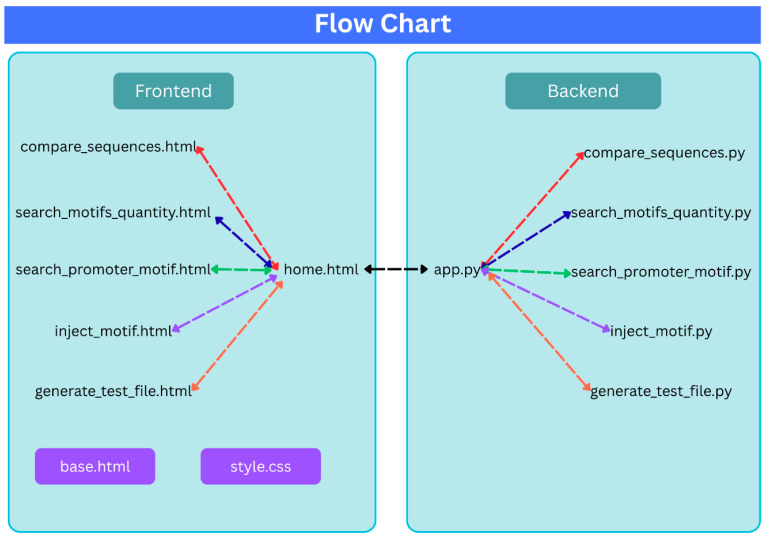
SW structure of the application. On the right side of the figure, we can see the Backend part and its Python scripts. On the left side, we see the Frontend part with its functions.

Our main contribution to the community is a new QA, open-source SW tool, which has been developed for testing any synthetic DNA sequence that was produced by any codon optimizer application. During the research, tests and statistics were performed on various sequences. The tests can be performed by a script written in Python, consisting of two main parts, as drawn in Figure 2 and explained below:

- Backend—This part includes the logic for the various tests, including:
  1. Manipulations on sequences.
  2. SW interface with optimization tools.
  3. Searching for promoter motif in different positions in sequences.
  4. Insertion attempt of promoter motif in different positions in sequences.
- Frontend—This part presents the product to the client in the form of a server that opens and displays the results on the Web based on Hyper Text Markup Language (HTML) and Cascading Style Sheets (CSSs).

There are two ways to access the Web application:

1. Users—Running the application via an “.exe” file.
2. Developers—Running the application via Python.

The open source described in the Supporting Information (SI) implements an application that can be accessed using the two methods described above.

Since it is an open source and we envision a design process that will continue as a collaborative effort, it is important to highlight the design principles. As shown in Figure 2, the application is designed with the following emphasis:

1. Modular structure and division between Backend and Frontend.
2. For each option available on the home page of the Web, a separate Python script was written to enable a proper development and debugging process.
3. For convenience, the developer who wants to run the application will also be required to run only one script—“app.py”—which is responsible for the integration and combination of all the scripts written in the project.

**Figure 3:**
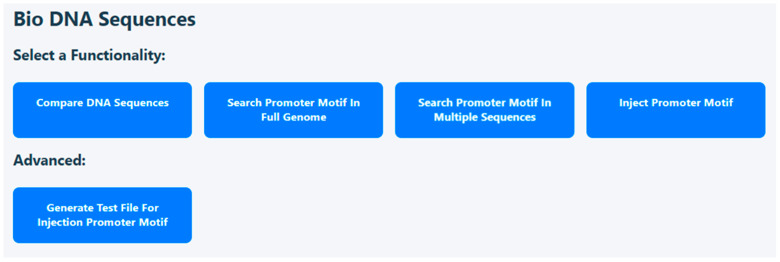
Current homepage of the Web application: Four basic functionalities and a single advanced option.

After running the application, you can access the local host URL on the Web. At this point, the application home page opens, which describes all existing functions that constitute the research results. Figure 3 shows the home page that will be opened when there is a login to the application. The user can compare DNA sequences and can search motifs in the full genome or in specific sequences. In addition, a promoter can be inserted into a sequence of choice. The advanced option is generating a test file for such insertion.

We focus below on three main functions that can be considered the core components of the algorithm. The first one is motif detection. The second one is about silent motif insertions. The third one is a function to secure your lab from unintended or malicious insertions.

#### 2.4.1. Antisense Promoter Motif Detection Algorithm

We use the motif detection function for two main purposes. Firstly, we use it to explore natural DNA sequences. It enables us to see if there is a natural evolutionary pressure to avoid certain sequences that should be avoided for any reason. Secondly, we use it to scan optimized sequences and detect problematic motifs like an antisense promoter. It enables us to test various optimization tools, prevent the usage of problematic sequences, and predict their impact in advance. As an example, we scanned for the “ATTATA” motif using a sliding 6-mer window. Motif frequencies were calculated per sequence and across the genome to establish baseline occurrence. Our SW is available with some high-level descriptions in the Supporting Information (SI).

#### 2.4.2. Motif Insertion Algorithm: Silent Insertion Probability

This tool tests the option of inserting a promoter motif into DNA sequences without changing the original protein sequence. The output refers both to the percentage of success and to the success for each specific sequence. It analyzes in which sequences the insertion attempt was successful, and in which cases and locations this happened for those sequences. Insertion simulations were conducted using an algorithm that iteratively substitutes synonymous codons in a target window to produce a reverse complement matching the target motif. We evaluated all possible codon combinations that encode the same amino acid chain while forming “TATAAT” on the antisense strand.

#### 2.4.3. Motif Defense Module: An SW Tool to Protect Your Lab

To detect potential insertions, we implemented a screening function that analyzes whether synonymous substitutions could feasibly produce antisense motifs. This tool flags sequences at risk and estimates the number of substitutions required for successful insertions. Based on the SW provided, we can also suggest alternative sequences for motif insertion.

## 3. Results

We present the results of our algorithms for the large-scale publicly available data. These results reveal insights regarding natural sequences, as well as demonstrate their efficiency in the case of synthetic sequences.

### 3.1. Natural Occurrence of Antisense Promoter Motifs and Insertion Feasibility in Motif-Free Sequences

The first test focused on the natural occurrence of antisense promoters. It enables the identification of the evolutionary pressure to avoid each motif and can lead to a better understanding of codon bias in various organisms. Among the 484,741 coding sequences analyzed, 23,057 (4.76%) contained the reverse-complement motif “ATTATA” (Table 1). These sequences were excluded from subsequent insertion tests. The motif’s rarity supports the hypothesis that antisense promoters are naturally avoided in coding DNA. All other motifs in the table were detected much more often and very close to the theoretical random calculation. Please note that the motif “ATACCT” can also lead to the “−10” motif, which is aligned with its low probability.

In the remaining 461,684 motif-free sequences, silent motif insertion was achievable in 356,797 cases (77.28%) through synonymous substitutions. Three simple examples of such insertions for SW validation are shown in Table 2.

### 3.2. Comparison with Random k-mer Frequencies

We calculate the probability of each insertion using the following procedure, demonstrated here for a specific 6-mer motif, to determine the probability of finding the antisense promoter motif, “ATTATA”, within a randomly generated sequence composed of amino acid codons. The probability calculations are based on the following naïve assumptions:

- All amino acids appear with equal probability in the sequence.
- No specific codon usage bias.

To find the probability of obtaining the motif randomly, it is necessary to determine how this sequence can be formed across different codon alignments. There are three frame shifts to be analyzed:

- Case 1: Two Complete Codons: “ATT”–“ATA”
- Case 2: Across Three Codons: “_ _ A”–“TTA”–“TA _”
- Case 3: Across Three Codons: “_ AT”–“TAT”–“A _ _”

For each of these cases, it is required to calculate the probability based on amino acid distributions.

For case 1:

“ATT” is a codon for Ile (I). “ATA” is also a codon for Isoleucine Ile (I). Because, according to codon usage bias, Ile has three codons (ATT, ATC, ATA), the probability of selecting “ATT” or “ATA” is 1/3 each. Since we assume that the probability of getting each amino acid is equal, the probability for each amino acid is 1/20. Therefore, the overall probability in this case will be calculated as follows:

(1) $$P_{Case1}=P\left(ATT,ATA\right)=P\left(ATT\right)\cdot P\left(ATA\right)=\left(\frac{1}{20}\cdot \frac{1}{3}\right)\cdot \left(\frac{1}{20}\cdot \frac{1}{3}\right)=2.78\cdot 10^{-4}$$

For case 2:

The amino acids with a codon ending with “A” are listed in Table 3. We can now calculate the probability of a codon ending with “A”:

(2) $$P\left(A_{end}\right)=\frac{1}{20}\cdot \left(\frac{1}{4}+\frac{1}{2}+\frac{1}{4}+\frac{1}{4}+\frac{1}{3}+\frac{1}{2}+\frac{1}{4}+\frac{1}{3}+\frac{1}{2}+\frac{1}{4}+\frac{1}{3}+\frac{1}{6}\right)=\frac{47}{240}$$

The next probability to calculate is for “TTA”, which is a codon for Leu (L). Because, according to codon usage bias, Leu (L) has 6 codons, the probability of selecting “TTA” is as follows:

(3) $$P\left(TTA\right)=\frac{1}{20}\cdot \frac{1}{6}=\frac{1}{120}$$

The only amino acid that starts with “TA” is Tyr (Y). Since we assume that the probability of getting each amino acid is equal, the probability will be as follows:

(4) $$P({TA}_{start})=\frac{1}{20}$$

Therefore, the overall probability in this case would be calculated as follows by considering the three probabilities found above:

(5) $$P_{case2}=P\left(A,TTA,TA\right)=P\left(A\right)\cdot P\left(TTA\right)\cdot P\left(TA\right)=\frac{47}{240}\cdot \frac{1}{120}\cdot \frac{1}{20}=1.63\cdot 10^{-4}$$

For case 3:

The amino acids that end with “AT” are listed in Table 4. Based on this table, we calculate the probability:

(6) $$P\left({AT}_{end}\right)=\frac{1}{20}\cdot \left(\frac{1}{2}+\frac{1}{2}+\frac{1}{2}+\frac{1}{2}\right)=\frac{1}{10}$$

The codon “TAT” is for Tyr (Y). Since Leu (L) has 2 codons, the probability of selecting TAT is as follows:

(7) $$\;P\left(TAT\right)=\frac{1}{20}\cdot \frac{1}{2}=\frac{1}{40}$$

Lastly, the amino acids that start with “A” are shown in Table 5. It yields the following calculation:

(8) $$P\left(A_{start}\right)=\frac{1}{20}\cdot \left(\frac{1}{2}+\frac{1}{2}+\frac{1}{2}+\frac{1}{2}+\frac{1}{4}+\frac{1}{3}\right)=\frac{31}{240}$$

Therefore, the overall probability in this case would be calculated as follows by considering the three probabilities found above:

(9) $$P_{frameshift[0]}=P\left(AT,TAT,A\right)=P\left(AT\right)\cdot P\left(TAT\right)\cdot P\left(A\right)=\frac{1}{10}\cdot \frac{1}{40}\cdot \frac{31}{240}=3.23\cdot 10^{-4}$$

The overall probability would be to test the union of the three cases:

(10) $$\;P_{total}=\frac{2.77\cdot 10^{-4}+1.63\cdot 10^{-4}+3.23\cdot 10^{-4}}{3}=2.54\cdot 10^{-4}$$

For comparison, the theoretical probability of randomly finding a six-nucleotide sequence assuming an equal distribution of nucleotides is as follows:

(11) $$\;P_{random}={\left(\frac{1}{4}\right)}^{6}=2.44\cdot 10^{-4}$$

These two different probability calculations are unsurprisingly almost equal.

We can now revisit the results of Table 1 for various 6-mers. Our control analysis using random 6-mers revealed that sequences like “GCCATC” and “TGGCAT” occurred at frequencies up to 3× higher than “ATTATA” and very close to the random calculation. It confirms the underrepresentation of certain motifs and likely selective pressure to avoid antisense promoter formation. The only random 6-mer that is relatively close to “ATTATA” is “ATACCT”, which has only 2 times higher probability. This specific sequence seems to create a sequence close enough to the “−10” site promoter motif for *E.coli*.

## 4. Discussion

Our findings reveal a significant and underrecognized vulnerability in codon optimization pipelines. While these tools aim to improve gene expression by refining synonymous codon usage, they unintentionally enable the creation of functional motifs on the antisense strand, such as bacterial promoter-like sequences. The widespread feasibility of silently inserting “ATTATA” across coding regions, with no effect on protein output, underscores this threat.

Biological implications include unintended transcriptional activity, interference with native gene regulation, and synthetic noise in engineered circuits. From a cyber-biosecurity perspective, this opens the door to adversarial attacks where malicious motifs are embedded into synthetic constructs—a form of sequence-level “backdoor” akin to steganography in SW systems.

Current design tools often ignore complementary strand effects. A defense module that can be easily implemented based on our detection algorithm should be incorporated into biofoundry pipelines. Future work can easily expand to other motif types (e.g., riboswitches, terminators), eukaryotic systems, and automated prevention mechanisms using AI-guided codon selection.

We envision the open-source code provided here becoming the basis of a much bigger set of QA tools that will be used by every lab before ordering DNA to decrease the probability of experimental failures. In addition, this set of tools can assist biologists in understanding evolutionary pressure to avoid certain motifs, leading to a better understanding of the genetic code.

## 5. Conclusions and Future Research Directions

Codon optimization has become indispensable in synthetic biology, yet our study highlights that it also presents hidden risks. Antisense promoter motifs such as “ATTATA” can be introduced without altering protein sequence in over 77% of motif-free sequences. We propose a framework for motif detection, insertion simulation, and defense screening strategy. Integrating such tools into standard codon optimization workflows is essential to safeguard biotechnology and basic biological research from inadvertent or malicious motif insertion.

Future research directions based on the open source code provided include the following:

1. Dive deeper into understanding the genetic code and various motifs with probabilities very different than random (both much higher and much lower).
2. Developing more QA tools to minimize the potential damage of synthetic DNA sequences that might encode surprises for biologists using these tools.
3. Identify other bioinformatics tools that should be examined within this framework to ensure their high quality and minimize their damage potential.

## Figures and Tables

**Table 1:** Scanning 484,741 proteins (546,462,945 bp) for 6-mer motifs. The −10 antisense motif is found in much lower probability than randomly generated motifs. One of the random motifs (“ATACCT”) could also serve as a −10 antisense motif.

The Sequence	Probability by Nucleotides $(P\sim 10^{-4}$)	Probability by Sequences $(P\sim 10^{-1}$)
ATTATA (motif)	${4.64\cdot 10}^{-5}$	${4.76\cdot 10}^{-2}$
GCCATC	${3.02\cdot 10}^{-4}$	${2.73\cdot 10}^{-1}$
CATTGC	${4.27\cdot 10}^{-4}$	${3.54\cdot 10}^{-1}$
TGGCAT	${1.22\cdot 10}^{-4}$	${1.26\cdot 10}^{-1}$
ATACCT	${8.92\cdot 10}^{-5}$	${8.85\cdot 10}^{-2}$
CGATCG	${1.85\cdot 10}^{-4}$	${1.67\cdot 10}^{-1}$

**Table 2:** Example of successful insertions of the promoter motif in the complementary strand. The DNA encodes a short peptide used as an SW validation case. All three insertions are found as expected. The first one requires only one change in the 3rd nucleotide of the first codon “AAC” 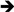 “AAT”. The other two insertion cases also generate the −10 motif in the antisense strand. The second case requires two changes, and the third case only one synonymous change.

Predicted DNA		Protein Sequence		Expected Case		
AACTATATGATCATTGCTTTATAT		NYMIIALY		[(case 3: start in codon 1), (case 1: start in codon 4), (case 2: start in codon 6)]		
**Original DNA**	**Modified DNA**	**# Insertions**	**Changes**		**Existing Motif**	**Cases**
AACTATATGATCATTGCTTTATAT	AATTATATGATTATAGCATTATAT	3	[(‘Insertion_1’, 2, ‘C’, ‘T’), (‘Insertion_2’, 11, ‘C’, ‘T’), (‘Insertion_2’, 14, ‘T’, ‘A’), (‘Insertion_3’, 17, ‘T’, ‘A’)]		FALSE	[3, 1, 2]

**Table 3:** The amino acids that end with “A”. The number of all optional codons for each amino acid is shown in the second column. The third column counts how many of them end with “A”. The probability in the last column is the division of the previous two.

c	Optional Codons	Codons That End in A	Probability
Gly (G)	4	1	1/4
Glu (E)	2	1	1/2
Ala (A)	4	1	1/4
Val (V)	4	1	1/4
Arg (R)	6	2	1/3
Lys (K)	2	1	1/2
Thr (T)	4	1	1/4
Ile (I)	3	1	1/3
Gln (Q)	2	1	1/2
Pro (P)	4	1	1/4
Leu (L)	6	2	1/3
Ser (S)	6	1	1/6

**Table 4:** The amino acids that end with “AT”. The number of all optional codons for each amino acid is in the second column. The third column counts how many of them end with “AT”. The probability in the last column is the division of the previous two.

Name of Amino Acid	Optional Codons	Codons That End in A	Probability
Asp (D)	2	1	1/2
Asn (N)	2	1	1/2
His (H)	2	1	1/2
Tyr (Y)	2	1	1/2

**Table 5:** The amino acids that start with “A”. The number of all optional codons for each amino acid is in the second column. The third column counts how many of them start with “A”. The probability in the last column is the division of the previous two.

Name of Amino Acid	Optional Codons	Codons That End in A	Probability
Arg (R)	2	1	1/2
Ser (S)	2	1	1/2
Lys (K)	2	1	1/2
Asn (N)	2	1	1/2
Thr (T)	4	1	1/4
Ile (I)	3	1	1/3

## Data Availability

The data supporting the findings of this study includes publicly available protein and gene annotation data obtained from the UniProt database, as well as genomic sequences retrieved from the NCBI database. In addition, synthetic DNA sequences were generated programmatically for validation and testing purposes. No sensitive or personal data was used. All scripts and algorithms developed for data generation, motif insertion, and motif analysis were created by the authors as part of the research and are available from the corresponding author upon reasonable request. No additional datasets were generated.

## Supplementary Materials

The following supporting information can be downloaded at: https://www.mdpi.com/article/10.3390/microorganisms14030638/s1, Supporting Information: Codon Insertion Algorithm for Targeted Antisense Motif Insertion: Mathematical Modeling and Empirical Analysis with ATTATA.
