# Supplementary material for: Unintended Creation or Insertion of Antisense Promoter Motifs During Codon Optimization: A Cyber-Biosecurity Risk
Source: Microorganisms. 2026 Mar 12;14(3):638. doi: 10.3390/microorganisms14030638 (PMC13029128; doi:10.3390/microorganisms14030638)
Supplement: Supplementary file 1 [file microorganisms-14-00638-s001.zip › microorganisms-4185835-supplementary.pdf]

Supplementary Information

Unintended Creation or Insertion of Antisense Promoter Motifs During Codon Optimization: A Cyber-Biosecurity Risk

Elad Carmi †, Roni Glikman † and Yuval Dorfan *

Faculty of Electrical Engineering, H.I.T.—Holon Institute of Technology, Holon 5810201, Israel; elad_carmi@hotmail.co.il (E.C.); r1glikman@gmail.com (R.G.)
* Correspondence: dorfany@hit.ac.il
† These authors contributed equally to this work.

1. Logic insertion and mathematical modeling

Given a target antisense motif M (e.g., ATTATA), our codon insertion algorithm searches for a valid gene sequence G′ such that:
- G′ encodes the same protein as G
- reverse_complement(G′) contains M

Let C(p) be the set of codons encoding the amino acid p. Let t = (p1, p2, ..., pn) be a protein sequence. The search problem becomes:

Find a sequence (c1, c2, ..., cn) such that ci ∈ C(pi) ∀i, and motif(reverse_complement(c1 + c2 + ... + cn)) = True

The problem is combinatorially large: If the average codon redundancy is r, and the motif spans k codons, then the total number of candidate windows is O(r^k).

2. Empirical analysis of 'ATTATA' motif insertion

An empirical analysis was performed on coding sequences extracted from the complete genome of *E. coli*. 4,108,805 nucleotides were scanned within coding regions to detect occurrences of the antisense promoter motif 'ATTATA':

(1) $P(\text{ATTATA}) = \frac{297}{4{,}108{,}805} = 7.23 \cdot 10^{-5}$

3. Example of silent insertion

Example: Inserting ATTATA across 3 codons
Original: GGC GCG AAC (Gly Ala Asn)

Modified: GGA GCT AAT
Both code for the same amino acids.
Reverse complement of modified: ATTATA — matching the bacterial promoter.

4. Application & code for biological analysis

There are several ways to access the application.

1. Github Repository (Recommended)
    - Users – Colab Notebook.
    - Developers – Clone Repository to local Windows environment.
2. Download Application & Full Product
    - Users – ’Bio_DNA_Sequences_WEB_App.zip’.
    - Developers – ’bio_dna_sequences-FullProductWithExe.zip’.
